# A meta-analysis of psychological factors in children with migraine and tension-type headache

**DOI:** 10.1186/1129-2377-14-S1-P20

**Published:** 2013-02-21

**Authors:** U Balottin, P Fusar Poli, C Termine, S Molteni, G Spada, G Nappi, F Galli

**Affiliations:** 1 Headache Science Center and Department of Child Neurology and Psychiatry. IRCCS ‘‘C. Mondino National Institute of Neurology’’, Italy; 2 Department of Psychosis Studies, Institute of Psychiatry, King's College London, UK; 3 Child Neuropsychiatry Unit, Department of Clinical and Biological Sciences, University of Insubria, Varese, Italy; 4 Headache Science Centre of the IRCCS ‘National Institute of Neurology C. Mondino’ Foundation, Pavia, Italy

## Introduction

Headache affects many children and adolescent causing disability. Many studies underline the role of psychological factors in children’s headache. A recent review [1] questioned the existence of psychological difficulties in migraine children, concluding that they don’t exhibit neither more psychological dysfunctions nor more psychiatric comorbidity then healthy controls. It is not clear how psychological factors effect on different kinds of headache. We wanted to clarify if there is a difference in the influence of psychological factors on migraine compared to healthy subjects and tension-type headache (TTH).

## Methods

We selected 10 studies that were comparable, had a control group, a sufficient sample size, sufficient data reporting and that used CBCL as a psycho-diagnostic tool. Internalizing and Externalizing disorders in different sub-types of headache and in healthy subjects were studied. Data were analyzed using Comprehensive Meta-Analysis Software version 2. The Hedges’g was adopted as a measure of effect size. We compared migraine patients vs controls, non-migraine patients vs controls and migraine vs TTH in 3 meta-analysis using Externalizing/Internalizing scales scores as a categorical moderator factor.

## Findings

Both migraine and TTH patients showed more psychopathology than healthy controls (respectively p <,001; p=0.0002). Both the sub-types showed more marked difference with the healthy controls at the Internalizing than at the Externalizing scale (TTH respectively p=0.009, 0=0.051). There was no significant difference between the two sub-types (migraine, TTH).

## Conclusion

Psychological factors influence headache in children, both migraine and TTH. We suggest to investigate this area and to treat children and adolescent in order to prevent a chronic evolution of the pain syndrome. CBCL may be a useful tool for a psychological evaluation.
